# Effect of chitosan edible coating containing anthocyanins and tea polyphenols on cold storage of chilled pork

**DOI:** 10.3389/fnut.2025.1546618

**Published:** 2025-05-07

**Authors:** Han Chen, Fang Zhang, Yi-xin Yang, Xiu-hua Meng, Xiao-qin Ding, Tun-yu Jian, Guan-ting Niu, Bei Tong, Ya-nan Gai, Han Lü, Jian Chen

**Affiliations:** ^1^Jiangsu Key Laboratory for the Research and Utilization of Plant Resources, Institute of Botany, Jiangsu Province and Chinese Academy of Sciences (Nanjing Botanical Garden Mem. Sun Yat-Sen), Nanjing, China; ^2^School of Pharmacy, Nanjing University of Chinese Medicine, Nanjing, China

**Keywords:** chitosan, anthocyanins, tea polyphenols, edible coatings, pork preservation

## Abstract

Preserving fresh meat has been a long-standing challenge due to microbial growth and lipid oxidation. In this study, chitosan (CS) active coatings loaded with blackberry anthocyanins (BA), tea polyphenols (TP), and their combination, designated as CS/BA, CS/TP, and CS/BA/TP, were applied for chilled pork preservation. Compared to CS coating alone, CS/BA/TP coating exhibited a 9.3% lower total viable count (TVC), a 45.5% reduction in thiobarbituric acid reactive substances (TBARS), and a 26.6% decrease in total volatile basic nitrogen (TVB-N) after 12 days of storage at 4°C. Furthermore, the combined use of TP and BA exhibited a synergistic effect, with the most pronounced impact observed in oxidative stability, while also improving microbial inhibition and maintaining color stability. These results highlight CS/BA/TP coatings as a promising natural preservative for extending chilled pork shelf life.

## Introduction

Preservation of fresh meat is a long-standing challenge due to its plentiful nutrients, which promote the growth of spoilage microorganisms and food-borne pathogens (19). Refrigeration storage is widely used to slow lipid oxidation and spoilage in meat. However, chilled meat remains susceptible to microbial contamination during storage and transport (20).

Synthetic petrochemical-based polymers have been widely used in meat packaging materials; however, due to their environmental pollution and the migration of toxic substances, biodegradable packaging materials have garnered significant interest in recent years (1). Edible films and coatings are thin layers used for food packaging and are made from edible components. Natural polysaccharides are excellent sources for edible coatings due to their biodegradable and inexpensive properties (2). Chitosan (CS) is obtained from chitin through deacetylation in an alkaline medium. It is a copolymer made up of β-(1–4)-2-acetamido-D-glucose and β-(1–4)-2-amino-D-glucose units, with the latter typically making up more than 60% of the composition. CS is an exceptional edible material due to its effective barrier properties against oxygen (O_2_) and carbon dioxide (CO_2_), along with excellent antimicrobial characteristics (21). However, its limited biological activity necessitates the incorporation of bioactive compounds, such as polyphenols, to enhance its functional properties (3).

In recent years, studies have confirmed that incorporating natural polyphenols into biodegradable food packaging films can boost their antioxidant and antibacterial properties, thereby enhancing their food preservation capabilities (22). Tea polyphenols (TP) are natural polyphenols derived from tea and belong to the flavanol group. In addition to being an effective food additive, TP functions as an antioxidant, antibacterial agent, and improving the performance of active food packaging films (4). Anthocyanins, a significant class of phenolic compounds found in nature, have been shown to enhance the physical and chemical properties of active films (23). Blackberry is a globally popular fruit rich in anthocyanins, with cyanidin-3-O-glucoside being the most prominent. Research has demonstrated that blackberry anthocyanins exhibit strong antioxidant and antibacterial properties (24). In our previous study, we found that combining blackberry anthocyanins (BA) and tea polyphenols (TP) effectively delayed lipid oxidation in edible oils during high-temperature processing (5). Recent studies have shown that chitosan coatings combined with TP and rosemary extract effectively extended the shelf life of large yellow croaker (6) and that chitosan-grape peel extract coatings reduced lipid oxidation and bacterial growth in beef during freeze–thaw cycles (7).

However, the effect of edible coatings containing TP and BA on meat preservation has not been thoroughly investigated. In this study, chitosan (CS) active coatings loaded with BA, TP, and a combination of both were applied for the preservation of pork. During storage, color parameters, pH value, total volatile basic nitrogen, total viable counts, and thiobarbituric acid reactive substances were measured to assess the effectiveness of the coatings in maintaining pork freshness. We hypothesize that integrating BA and TP into CS coatings will exhibit a synergistic effect, enhancing the antioxidant and antimicrobial properties of the coatings and extending the shelf life of chilled pork.

## Materials and methods

### Chemical and reagents

Chitosan powder (CS, ≧90 % degree of deacetylation) was obtained from Sangon Biotech (Shanghai) Co., Ltd. (Shanghai, China). Blackberry anthocyanins (BA) was prepared in our previous work. Tea polyphenol (TP, ≧98 % purity) was purchased from Yuanye Bio-Technology Co., Ltd. (Shanghai, China). The chemicals used in this study were analytical grade and obtained from Macklin Biochemical Co., Ltd. (Shanghai, China).

### Preparation of chitosan coatings and pork samples

Based on our preliminary study, CS (2.25% w/v) was dispersed in a 1% (v/v) hydrochloric acid (HCl) solution under magnetic stirring at room temperature for 2 h to obtain CS solution, which appeared transparent and slightly yellowish. BA (0.44 wt%) and TP (6.67 wt%), dissolved in water, were individually added to the CS solution under magnetic stirring at room temperature for 1 h, based on the total weight of CS, to obtain the CS/BA and CS/TP solutions, respectively. The CS/BA solution exhibited a reddish-purple hue, while the CS/TP solution appeared light brown. The CS/BA/TP solution were prepared by adding mixed BA (0.44 wt%) and TP (6.67 wt%) solution to the CS solution under magnetic stirring at room temperature for 1 h, resulting in a dark purplish-brown solution. Subsequently, glycerol (1% v/v) was added under magnetic stirring to obtain the final coating solutions.

The devices and work surfaces were sterilized with 75% ethanol and exposed to ultraviolet (UV) light for 0.5 h before the experiment. The fresh pork tenderloin purchased from a local supermarket (Nanjing, China) was cut into steaks under sterile conditions and divided into five groups. The pork samples were individually dipped into the prepared coating solutions (CS, CS/BA, CS/TP, CS/BA/TP) for 60 s, then air-dried to form a coating. Uncoated pork samples were treated as control (Control). All samples were placed in sterile trays, sealed with plastic wrap and stored at 4 ± 1°C with a relative humidity of 80% in darkness for 12 days.

### Color parameters

The color parameters [L^*^ (lightness), a^*^ (redness), and b^*^ (yellowness)] of the pork samples were measured at three random locations on each surface of the sample at 4 ± 1°C using a portable spectrophotometer (NR10QC, Shenzhen ThreeNH Technology Co., Ltd., China). The total color difference (ΔE_t_) was calculated according to Equation 1:


(1) $$\text{Δ}E_{t}=\sqrt{{\left(L^{*}-L_{0}\right)}^{2}\;+\;{\left(a^{*}-a_{0}\right)}^{2}+\;{\left(b^{*}-b_{0}\right)}^{2}}$$

where *L*_0_, *a*_0_ and *b*_0_ are the color parameters of day 0. L^*^, a^*^ and b^*^ are the color parameters of the samples at different storage times (3, 6, 9, and 12days) (8).

### pH value

The pH was determined according to the Chinese national standard GB 5009.237-2016 (27). One gram of the sample was added to 10 g of 0.1 M potassium chloride solution. Then the mixture was homogenized, and the pH was measured using a digital pH meter (FiveEasy Plus, METTLER TOLEDO, USA). Three measurements were taken on each meat sample, and the average was recorded as the pH of the meat sample. Before use, the pH meter was calibrated with standard buffer solutions at pH 4.00, 6.86 and 9.18, respectively.

### Total volatile basic nitrogen (TVB-N) value

TVB-N value of the pork was measured according to the Chinese national standard GB 5009.228-2016 (26). Ten gram of sample was homogenized with 50 mL of distilled water and filtered after impregnating for 30 min. One milliliter of boric acid solution and 1 drop of mixed indicator were added into the central chamber of the diffusion dish. Simultaneously, the 1 mL of filtrate and 1 mL of saturated sodium carbonate solution were added into the outer chamber of the diffusion dish. All solution were mixed evenly and stored in the sealed diffusion dish at 37 ± 1°C for 2 h. The solution in the central chamber was then titrated with hydrochloric acid. The TVB-N value of each sample was determined based on triplicate measurements, and volatile nitrogen bases were calculated in terms of milligrams of nitrogen per 100 g of the sample using the following equation:


$$\begin{array}{l} \text{TVB}-\text{N }\left(\text{mg}/100\text{g}\right)\;=\text{ C }\times \;\left(\text{V}-{\text{V}}_{0}\right)\;\times \;14\times 5000/\text{W} \end{array}$$

Where V is the volume of hydrochloric acid consumed, V_0_ is the volume of hydrochloric acid consumed in the titration process of blank (ml), W is the weight of meat sample (g), and C is the concentration of hydrochloric acid (g/mol).

### Thiobarbituric acid reactive substances value

The lipid oxidation of pork was evaluated by measuring TBARS according to the Chinese national standard GB 5009.181-2016 (25). Briefly, 5 g of ground sample was blended and homogenized with 50 mL of 7.5% trichloroacetic acid containing 0.1% EDTA, and stirred at 50°C for 30 min using a magnetic stirrer. After cooling to room temperature, the homogenate was filtered with double-layer quantitative slow filter paper. The thiobarbituric acid (TBA) reagent was prepared by dissolving 0.2883 g of TBA in 100 mL of distilled water to obtain a 0.02 M solution. Then, 5 mL of filtrate was transferred to a screw cap test tube, and after adding with 5 mL of 0.02 M TBA solution, the solution was reacted in a water bath at 90°C for 30 min and analyzed optically at 532 nm using a spectrophotometer. The content of malondialdehyde in the sample was calculated according to the standard curve of malondialdehyde, referred to as the TBARS value, and expressed as milligrams of malondialdehyde per kilogram of sample (mg MDA/kg).

### Microbial analysis

Total viable count (TVC) was measured by the plate count agar (PCA) method to assess microbial growth in samples at 0, 3, 6, 9 and 12 days of refrigerated storage duration. Meat samples (5 g) were homogenized with 45 mL of 0.1% peptone water. Ten-fold serial dilutions of each sample homogenate were made and applied to agar plates, which were then incubated at 37 °C for 48 h. The results were recorded as log CFU (colony-forming units) per gram of meat sample.

### Statistical analysis

Statistical analysis was performed using GraphPad Prism version 8 (GraphPad Software, Inc., CA, USA) and Origin version 2019b (OriginLab Corporation, USA). Each independent analysis was performed in triplicate (*n* = 3) for which results were expressed as the mean ± standard deviation. Date collected were analyzed using two-way-analysis of variance (ANOVA; treatment × storage) followed by Duncan's test to compare differences between means of parameters at the 5% significance level. Differences were considered significant at *p* < 0.05.

In this study, the independent variables were coating treatment (CS, CS/BA, CS/TP, and CS/BA/TP) and storage time (0, 3, 6, 9, and 12 days). The dependent variables included color parameters (L, a, b^*^), pH, total volatile basic nitrogen (TVB-N), total viable counts (TVC), and thiobarbituric acid reactive substances (TBARS), which were used to assess the effectiveness of the coatings in preserving pork quality.

## Results and discussion

### Color parameters

**Figure 1:**
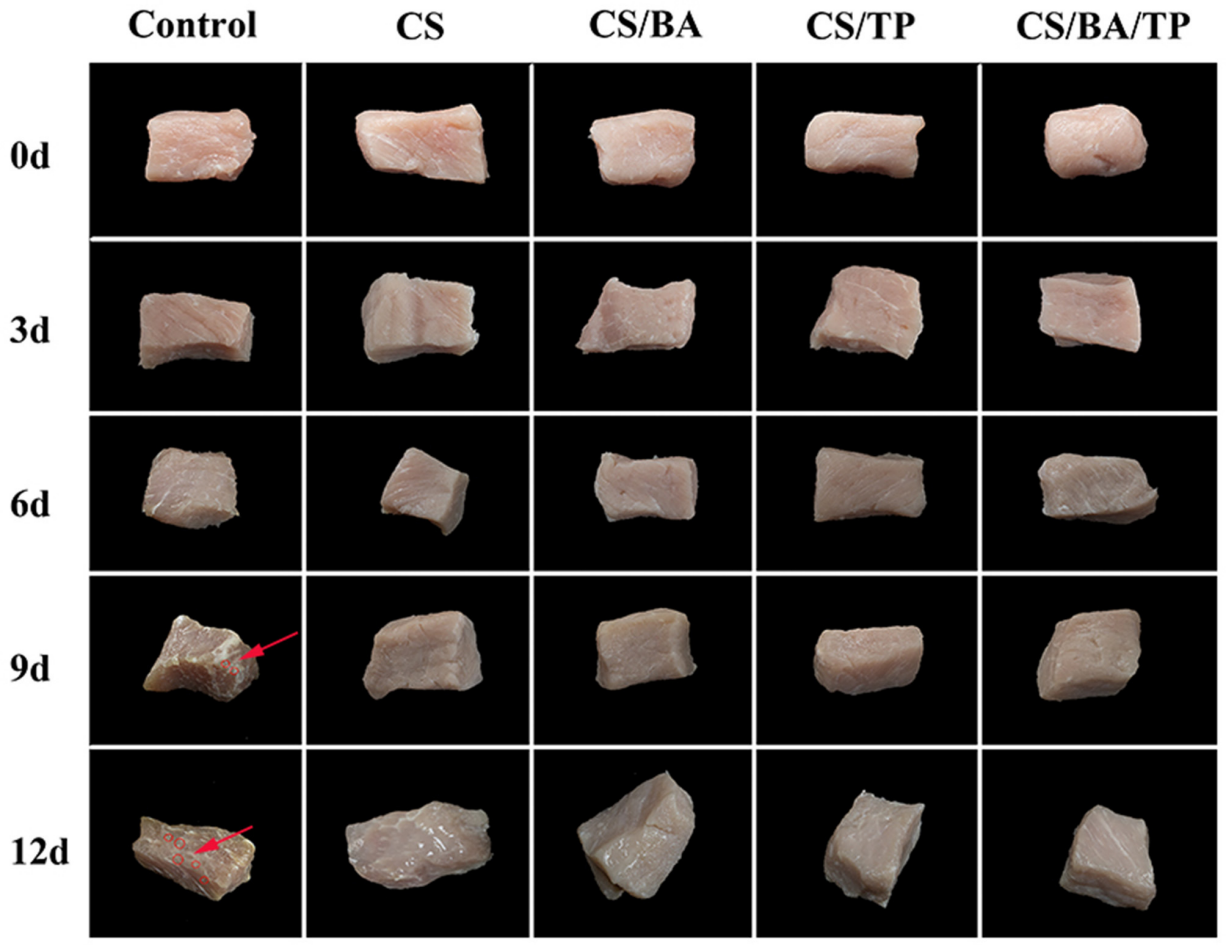
Changes of appearance of pork samples with different packaging groups during cold storage (4°C; bacterial colonies indicated by red arrow).

Meat color is an important indicator for consumer choice. As shown in Figure 1, with increased storage time, the color of control pork samples darkened, and water loss and dry shrinkage occurred. Bacterial colonies appeared on day 9, with more colonies appearing on day 12. In comparison, the changes in the different coating groups were less significant, and no obvious colonies appeared.

**Figure 2:**
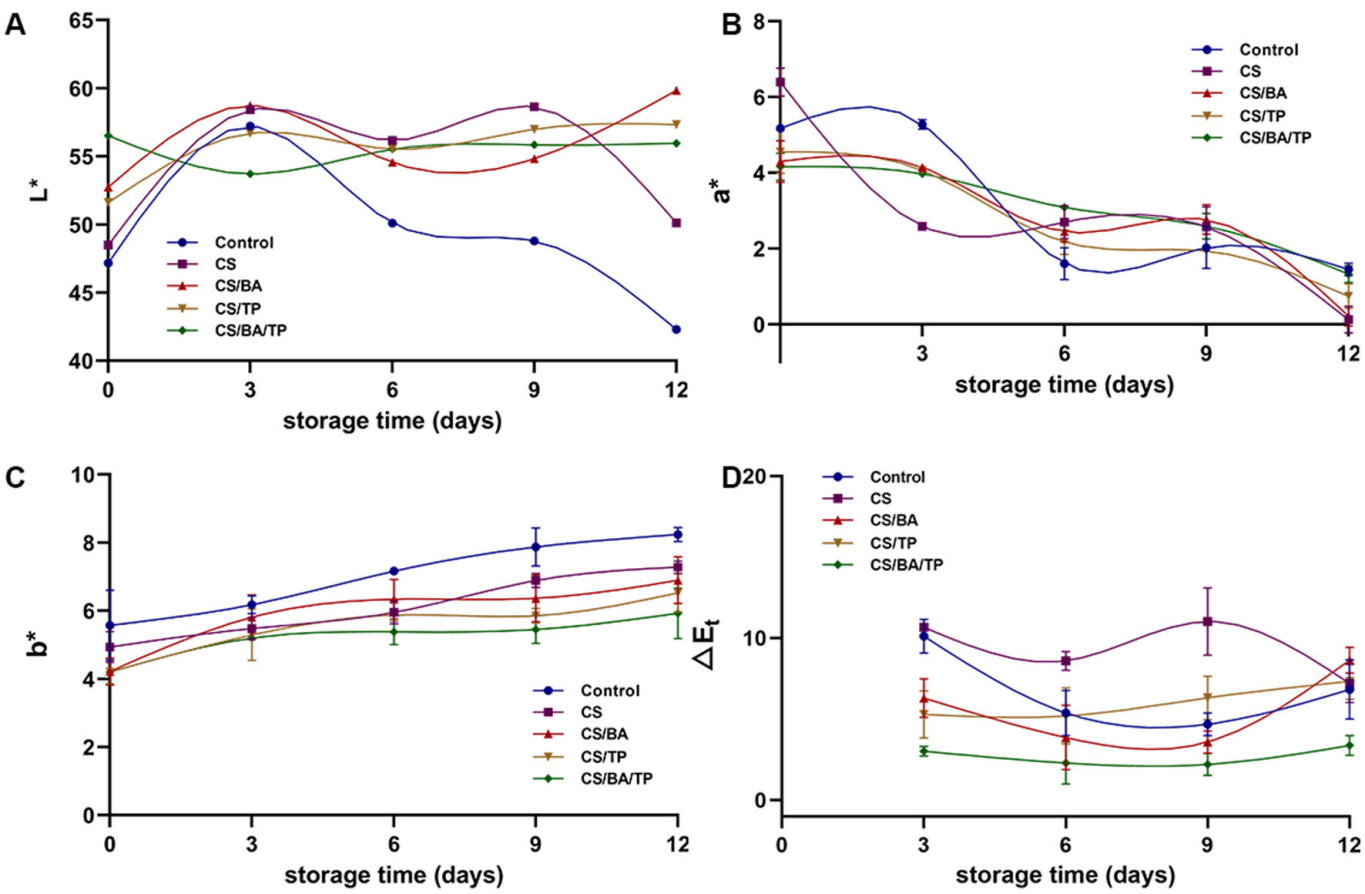
Changes of color parameters including L*(A), a*(B), b* values **(C)** and ΔE_t_ **(D)** of pork samples with different packaging groups during cold storage (4°C).

At day 0, after coating with different films, the brightness (L^*^ value) of chilled pork samples increased (Figure 2A). The L^*^ value of the control group increased on day 3 and then decreased rapidly, dropping from 57.22 ± 1.61 to 42.30 ± 1.38 by day 12. The alteration in L^*^ value in the CS coating samples were similar to that in the control group, but the extent was lower. The changes in L^*^ values for the CS/BA, CS/TP, and CS/BA/TP coating samples were smaller, with the L^*^ values in the CS/BA/TP samples remaining relatively stable (*p* > 0.05) over 12 days. This indicates that the CS/BA/TP coating can help maintain the light value of pork.

Compared with the control group, coating with CS increased the redness index (a^*^ values) of pork (Figure 2B), while the a^*^ values of the CS/BA, CS/TP, and CS/BA/TP coating samples were lower than those of the control sample on day 0, indicating that the addition of BA and TP may influence the redness index of the meat. The a^*^ values of all pork samples decreased significantly with storage time, suggesting that these coatings have no obvious effect on maintaining the red color of pork.

With the increase in storage time, the b^*^ value of the control group samples increased gradually (Figure 2C), which is consistent with the meat color changing from red to brown. Lipid oxidation in meat may transform white fat into a yellow color. The formation of yellow pigments affected by lipid oxidation in meat is related to the non-enzymatic browning reactions of lipid oxidation products (28). All coatings decreased the yellowness b^*^ values of pork compared to control samples on day 0. In comparison, the increase in b^*^ values of the coating samples ware smaller than that of the control group, with the CS/BA/TP group showing the least increase. The CS coating could significantly inhibit the increase of yellowness value, which may be related to its oxygen barrier ability, thereby retarding lipid oxidation (21). The addition of BA or TP increased the effectiveness of CS in inhibiting the increase in b^*^ value, with the simultaneous addition of both BA and TP showing the best effect. This improvement may be due to the antioxidant properties of BA and TP, which may enhance the anti-lipid oxidation effects of the CS coating.

The total color difference (ΔE_t_) between days (3 vs. 1, 6 vs. 1, 9 vs. 1 and 12 vs.1) for five batches (Control, CS, CS/BA, CS/TP, and CS/BA/TP) was calculated (Figure 2D). Most remarkable differences were observed on day 9 vs. 1 (CS > CS/TP> Control > CS/BA > CS/BA/TP). The ΔE_t_ value of the CS/BA/TP group was always the lowest among all groups, which indicated that the CS/BA/TP coating promoted the color stability of pork during storage.

### Microbiological analyses

**Figure 3:**
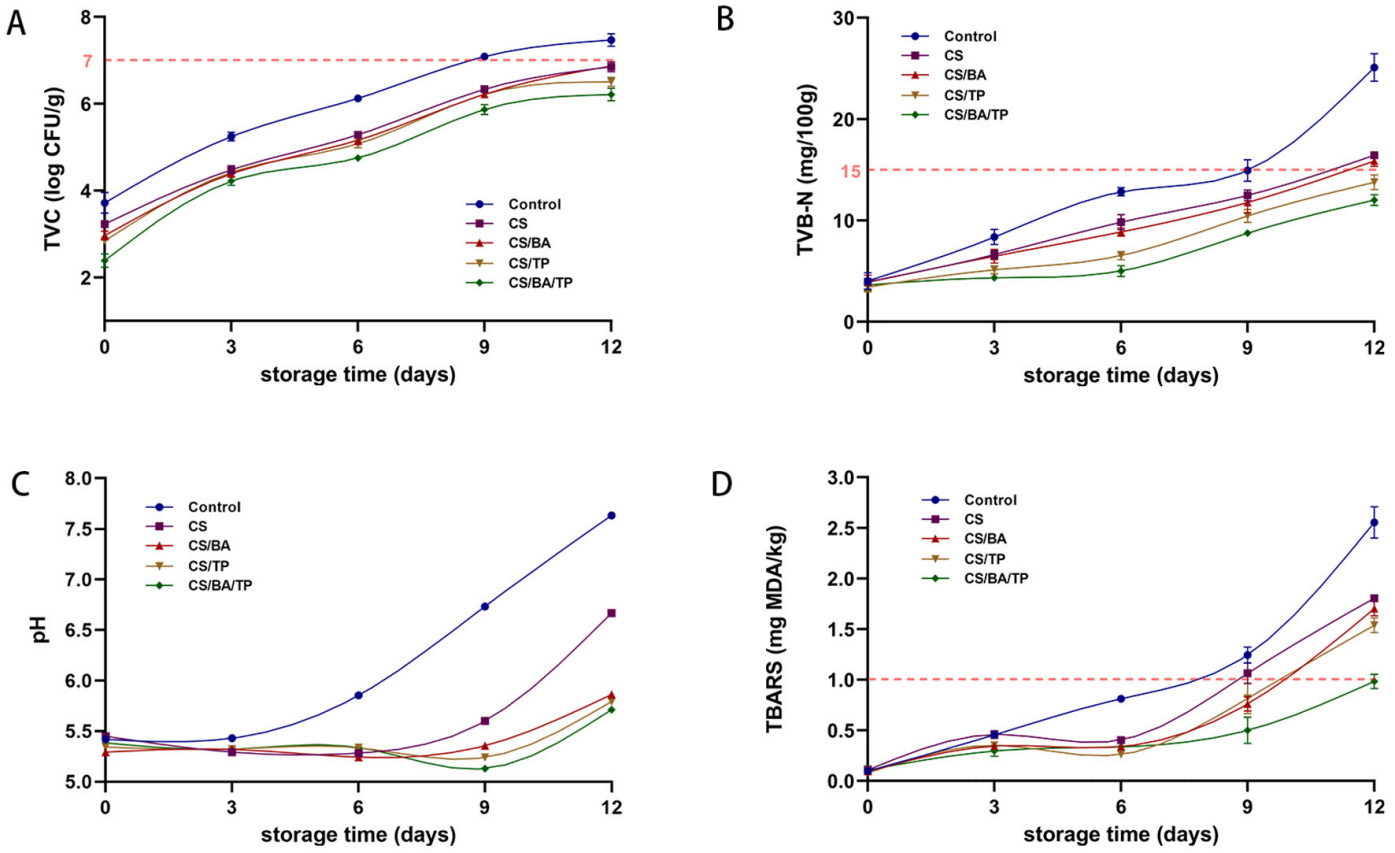
Changes of TVC **(A)**, TVB-N **(B)**, pH **(C)**, TBARS **(D)** values of pork samples with different packaging groups during cold storage (4°C).

Microbiological growth is the most important factor influencing the changes that cause spoilage in meat. The total viable count (TVC) is an important microbial index to evaluate the hygienic quality, safety and spoilage of meat. As shown in Figure 3A, the TVC values of all coating groups were significantly lower than those of the control sample at 0 days. Throughout the storage period, the TVC values of all samples gradually increased, but the coating groups consistently exhibited significantly lower TVC values compared to the control group (*p* < 0.05). After the 9-day storage period, the TVC of control group samples were 7.09 ± 0.04 log CFU/g, up to the threshold defined for the quality of pork (29). While all of the coating samples were below this threshold after the 12-day storage period, indicating that coating may contributed to extend the meat shelf-life by limiting the oxygen availability for microbial growth (9). At the end of the storage period, the TVC of the CS/BA/TP coating was 6.21 ± 0.15 log CFU/g, significantly lower than that of CS, CS/BA, and CS/TP (*p* < 0.05). Similar to other composite chitosan coatings for preservation, the incorporation of one or more active substances demonstrated superior antibacterial performance (4, 10).

The observed antimicrobial effects of the coatings align with previous reports on chitosan (30), and both TP and BA also demonstrate antibacterial properties (24, 31). The peptidoglycan layer and teichoic acid are key components of Gram-positive bacterial cell walls, and TP has been reported to disrupt the peptidoglycan structure, leading to bacterial inhibition (11). Furthermore, studies suggest that polyphenol-based compounds can enhance bacterial damage when combined with other bioactive agents, demonstrating a synergistic antibacterial effect (9). This experiment further confirmed the antibacterial effects of TP and BA in meat preservation. This result is consistent with findings from other studies on pork preservation (4, 10, 12). The active coating loaded with both BA and TP was more effective in reducing the TVC of pork during storage than other coatings, suggesting a synergistic effect in preventing microbial spoilage.

### TVB-N value

During meat spoilage, the degradation of proteins and other nitrogen (N)-containing compounds causes the accumulation of organic amines, commonly known as total volatile basic nitrogen (TVB-N), which are toxic and can lead to considerable changes in color and flavor, seriously affecting the quality of the meat. Therefore, TVB-N content is an important factor in evaluating meat quality (32).

As shown in Figure 3B, all groups exhibited a gradual increase in TVB-N with extended storage time, which may be related to the proliferation of microorganisms that accelerate the decomposition of pork proteins and produce amines (33). Throughout the entire storage period, the TVB-N value of the control showed a significant increasing trend. In contrast, all coatings significantly delayed the increase in TVB-N value (*p* < 0.05) compared to the control samples. According to the results of the microbiological analyses above, CS coating may suppress microbial growth, and the addition of BA and TP can enhance this effect. All of these factors may lead to lower production of TVB-N in meat. It was suggested that a TVB-N value of 15 mg/100 g may serve as the upper threshold for acceptable freshness of pork. The TVB-N value of the control sample reached this threshold on day 9, indicating that its shelf life is no more than 9 days. TVB-N values of the CS and CS/BA coating samples did not reach the threshold until day 12. However, the TVB-N values of the CS/TP and CS/BA/TP coatings were still below the threshold value on day 12, indicating that their shelf life was extended to 12 days.

### pH values

As shown in Figure 3C, the pH value of the control group significantly increased throughout the entire storage period. Compared to the control group, there was no significant change in pH in any of the coating groups within the first 6 days. The pH of the CS group significantly increased on day 9, while there was no significant change in the CS/BA group, and the CS/TP and CS/BA/TP coating groups showed a slight decrease in pH. All coating groups exhibited an increase in pH by the 12th day, but the pH of the CS/BA and CS/TP or CS/BA/TP coating groups was significantly lower than that of the CS coating group. The pH increase in the control group can be attributed to protein denaturation and the buildup of organic amines, such as ammonia, amines, and trimethylamine, which are generated during the degradation of amino acids through autolytic or microbial processes (34). Studies have shown that chitosan-gelatin coating can create a protective barrier on the surface of pork, reduce the exchange of substances between the meat and the surrounding environment, and slow down the propagation of microorganisms and the decomposition rate of proteins to some extent (4). The differences in pH trends among the coating groups may be attributed to the distinct mechanisms of BA and TP. BA contains organic acids, which may contribute to a direct pH-lowering effect (13), whereas TP exhibits stronger antimicrobial properties, effectively inhibiting microbial metabolism and acid production, leading to a more gradual pH decline (14).

The pH of all coating samples in this experiment is significantly lower than that of the control group, which supports the previous researches. Moreover, the pH growth rates of the CS/BA, CS/TP, and CS/BA/TP groups were significantly lower than that of the CS group (*p* < 0.05). This indicates that the addition of BA, TP, and BA/TP effectively inhibited the increase in pH of chilled pork, likely due to the further inhibition of protein decomposition and microbial reproduction in the meat by the addition of active substances. The coating with the combined addition of BA and TP had a synergistic effect in inhibiting the increase in pH of chilled pork.

### Lipid oxidation

Pork has a high fat content and is highly susceptible to lipid peroxidation. The resulting oxidative changes, including alterations in flavor, color, texture, and nutritional value are significant factors that contribute to quality deterioration during storage. Our previous research has demonstrated that BA and TP exhibit strong scavenging activity against ABTS and DPPH free radicals (5). TBARS assay was used to evaluate the lipid oxidation levels in different samples, with higher TBARS values indicating elevated levels of lipid oxidation. Studies have shown that the TBARS value of fresh meat should be controlled within 1.00 mg MDA/kg, as exceeding this threshold leads to noticeable oxidative deterioration and negatively impacts sensory quality (15). As shown in Figure 3D, the TBARS value of the control group increased rapidly with storage time. The TBARS values of the CS, CS/BA, and CS/TP coating groups showed a slow increase during the first 3 days and slightly decreased by the 6th day. This may be because CS, BA, and TP had not yet fully exerted their effects in the early stage of coating and were unable to fully inhibit the acceleration of the lipid oxidation process. After 3 days, the CS, CS/BA, and CS/TP coating solutions tightly bound to the pork samples, reducing oxygen permeability, and the antioxidant components began to take effect, slowing down the lipid oxidation process and leading to a decrease in TBARS values. After day 6, all coating groups exhibited a rapid increase in TBARS values, but the CS/BA/TP group maintained a significantly lower oxidation rate than the other groups. By the 9th day, both the control and CS groups had exceeded the limit of 1.00 mg MDA/kg, while the CS/TP, CS/BA, and CS/BA/TP groups remained below this limit. However, by the 12th day, all groups except the CS/BA/TP group had exceeded the limit of 1.00 mg MDA/kg, indicating that the coating with the combined addition of BA and TP had a synergistic effect in slowing lipid oxidation. This synergy may be attributed to the complementary antioxidant mechanisms of BA and TP, where BA donates hydrogen atoms to scavenge free radicals, while TP enhances oxidative stability through metal ion chelation and radical regeneration (16). Consequently, the CS/BA/TP coating effectively inhibited TBARS accumulation, significantly extending the shelf life of chilled pork to 12 days.

## Conclusion

In this study, chitosan (CS) active coatings loaded with blackberry anthocyanins (BA) and tea polyphenols (TP) and a combination of both were tested for their effects on pork preservation. Compared to the CS coating alone, the CS/BA/TP coating exhibited a 9.3% lower total viable count (TVC), a 45.5% reduction in thiobarbituric acid reactive substances (TBARS), and a 26.6% decrease in total volatile basic nitrogen (TVB-N) after 12 days of storage at 4°C. The combined addition of TP and BA demonstrated a synergistic effect, further enhancing antimicrobial and antioxidant properties. These results suggest that CS coatings incorporating TP and BA effectively extend the shelf life of chilled pork to 12 days, making them a promising natural preservation strategy.

While synthetic preservatives and modified atmosphere packaging (MAP) are widely used in the meat industry, natural bio-based coatings like CS/BA/TP provide a clean-label alternative with strong antimicrobial and antioxidant properties (17).

Although the use of BA and TP may slightly increase production costs, their ability to extend shelf life, reduce spoilage, and minimize reliance on synthetic additives could offset these costs. Furthermore, the ease of application and biodegradability of CS-based coatings align with the growing demand for sustainable and natural food preservation solutions, supporting their potential for commercial adoption (18).

## Data Availability

The original contributions presented in the study are included in the article/supplementary material, further inquiries can be directed to the corresponding authors.
